# Electron Pathways through Erythrocyte Plasma Membrane in Human Physiology and Pathology: Potential Redox Biomarker?

**Published:** 2007-09-17

**Authors:** Elena Matteucci, Ottavio Giampietro

**Affiliations:** Department of Internal Medicine, University of Pisa, Pisa, Italy

**Keywords:** Human erythrocyte, Na/H exchanger, Plasma membrane oxidoreductase, Type 1 diabetes, Diabetic nephropathy, Uremia

## Abstract

Erythrocytes are involved in the transport of oxygen and carbon dioxide in the body. Since pH is the influential factor in the Bohr-Haldane effect, pHi is actively maintained via secondary active transports Na^+^/H^+^ exchange and HC_3_^−^/Cl^−^ anion exchanger. Because of the redox properties of the iron, hemoglobin generates reactive oxygen species and thus, the human erythrocyte is constantly exposed to oxidative damage. Although the adult erythrocyte lacks protein synthesis and cannot restore damaged proteins, it is equipped with high activity of protective enzymes. Redox changes in the cell initiate various signalling pathways. Plasma membrane oxido-reductases (PMORs) are transmembrane electron transport systems that have been found in the membranes of all cells and have been extensively characterized in the human erythrocyte. Erythrocyte PMORs transfer reducing equivalents from intracellular reductants to extracellular oxidants, thus their most important role seems to be to enable the cell respond to changes in intra- and extra-cellular redox environments.

So far the activity of erythrocyte PMORs in disease states has not been systematically investigated. This review summarizes present knowledge on erythrocyte electron transfer activity in humans (health, type 1 diabetes, diabetic nephropathy, and chronic uremia) and hypothesizes an integrated model of the functional organization of erythrocyte plasma membrane where electron pathways work in parallel with transport metabolons to maintain redox homeostasis.

## Introduction

The erythrocyte is a highly specialized cell whose main functions are oxygen transport and the mediation of carbon dioxide transport (Siems et al. 2000). Glycolysis and the oxidative pentose phosphate pathway generate NADH and NADPH to reduce methemoglobin, which is being continuously produced, and the antioxidant glutathione, which is present in high concentrations. Most of the nonenzymatic antioxidant capacity of whole blood is likewise localized in the erythrocytes. Circulating red cells are mobile free radical scavengers and provide antioxidant protection to other tissues and organs. At the end of their life span (120 ± 4 days) the human erythrocytes become non-self cells and then are phagocytosized (Arese et al. 2005). Band 3 modifications, mostly due to oxidative insults, are presumed to lead to band 3 oligomerization, anti-band 3 binding, complement activation, opsonization, and phagocytosis. Despite increasing evidence in literature that plasma membrane oxidoreductases may have several functions, little data exists about the erythrocyte electron transfer systems in humans. The cell serves as a model to discuss the physiological and pathological implications of emerging evidence on the functional organization of plasma membranes and to highlight the potential role of transmembrane electron transfer as redox biomarker.

## Erythrocyte Energy Metabolism

The adult erythrocyte lacks the ability to synthesize proteins and cannot restore damaged proteins, but it is equipped with high activity of protective enzymes. Energy is needed to maintain hemoglobin (Hb) iron in the divalent form, transmembrane gradients of sodium and potassium, the sulfhydryl groups of enzymes and Hb in the reduced form, and the biconcave shape of the cell. The red blood cell (RBC) lacks citric acid cycle and extracts energy from glucose essentially by anaerobic glycolysis or Embden-Meyerhof pathway (Beutler, 1986). Branching of the metabolic flow after the formation of 1,3-diphosphoglycerate (Luebering-Rapaport pathway) determines both the rate of ADP phosphorylation and the concentration of 2,3-diphosphoglycerate whose levels are particularly sensitive to pH. The hexose monophosphate shunt reduces NADP^+^ to NADPH, i.e. the substrate for glutathione reductase to generate glutathione (GSH). Under normal steady-state conditions, 92% of glucose is metabolized by glycolysis and 8% through the hexose monophosphate shunt. Under oxidant conditions up to 90% of glucose can be metabolized along the hexose monophosphate shunt. Moreover, oxygenation-dependent interactions between Hb, cytoplasmic factors and cytoskeletal proteins may alter erythrocyte properties and metabolism (Barvitenko et al. 2004).

Among a number of hormone and cytokine receptors (Minetti and Low, 1997; Di Virgilio et al. 2001), erythrocytes express purinergic P2 receptors and at the same time readily release ATP. Therefore, they are an ideal “integrator unit” in the blood because they are both sensitive to ATP released by other blood cells and are able to modulate the function of circulating or endothelial cells by secreting this nucleotide.

## Erythrocyte Membrane Skeleton as a Membrane Scaffolding

**Figure 1:**
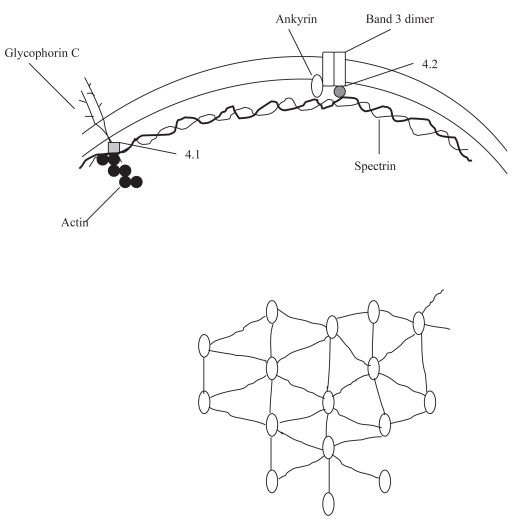
Schematic model of the organization of the cytoskeleton of human erythrocytes. The spectrin molecules form a mesh-like pattern (hexagonal) that is anchored to the membrane by ankyrin molecules.

RBC cytoskeleton maintains the biconcave shape of the erythrocyte, its reversible deformability and membrane structural integrity (Fig. 1). The spectrin oligomeric lattice binds to specific classes of integral membrane proteins through a number of accessory proteins (Bennett and Lambert, 1991; Mills and Mandel, 1994). The band 3 anion exchanger is the major integral protein. Its C-terminal cytoplasmic domain binds carbonic anhydrase II (CAII); the N-terminal cytoplasmic domain (cdb3) binds glycolytic enzymes, Hb, and hemichromes. A second site of attachment of the RBC membrane to the cytoskeleton is through the glycophorin C complex. The existence of the Rh protein complex has been also suggested (Bruce et al. 2003; Mohandas and An, 2006).

In cell membranes, local inhomogeneity in the lateral distribution of lipids and proteins is thought to exist in vivo, in the form of lipid rafts and in specific classes of proteins, specialized in signal transduction, cell-cell recognition, and vesicular trafficking. Lipid rafts are proposed to be cholesterol- and sphingolipid-rich domains that float in an environment of more fluid regions. Caveolae represent a subtype of lipid raft that form flask-shaped membrane invaginations containing the structural protein caveolin1 (Gaus et al. 2006; Ciana et al. 2005).

**Figure 2:**
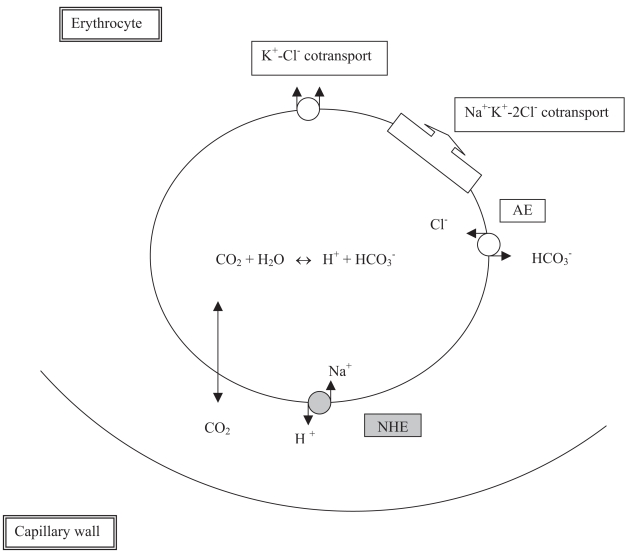
Summary of membrane-bound transport systems involved in ion fluxes between erythrocyte and blood. NHE: Na^+^/H^+^ exchanger. AE: Cl^−^/HCO_3_^−^ anion exchanger.

Sorting of biochemical pathways depends on the accumulation of proteins within different membrane-bound compartments, which exist as dynamic membrane systems rather as isolated entities. The membrane skeleton possesses characteristics predicted for a membrane protein-sorting machine (Gaus et al. 2006; Beck and Nelson, 1996). In addition to binding ankyrin, protein 4.1, protein 4.2, kinases, hemichromes, integral membrane proteins, and glycolytic enzymes (GE), the N-terminal cytoplasmic domain cdb3 associates preferentially with the deoxygenated state (T state) of Hb. Indeed, human cdb3 competes with 2,3-diphosphoglycerate for occupancy of the central cleft in the deoxyHb tetramer (Weber et al. 2004). The rate of glycolysis, the pentose phosphate pathway, and a number of ion transport pathways in erythrocytes have been shown to depend on the state of Hb oxygenation. On Hb deoxygenation, glycolysis accelerates, the pentose phosphate pathway declines, the activities of the Na^+^-K^+^-2Cl^−^ cotransporter and the Na^+^/H^+^ exchanger (NHE) rise (Fig. 2).

The glycolytic machinery in the human erythrocyte can compartimentalise ATP, allowing its direct consumption by ion pumps without release into the cytoplasm (Campanella et al. 2005). Phosphorylation of band 3 or deoxygenation of Hb can lead to release of GE from the membrane and accelerated glycolysis. Hb-cdb3 interaction could also mediate oxygen-sensitive ion transport involved in erythrocyte volume and pHi regulation.

## Erythrocytes, Oxygen Transport, and Oxygenation-dependent Signal Transduction Pathways

RBCs transport O_2_ to the tissues to support oxidative phosphorylation. Bohr revealed the influence of pH/CO_2_ on O_2_ binding (Jensen, 2004). The reciprocal Haldane effect (influence of O_2_ binding on H^+^/CO_2_ binding) has the same molecular origin. During oxygenation, tetrameric Hb undergoes a conformational change from the low affinity (tense, T) structure to the high-oxygenated (relaxed, R) structure that has a lower capacity for binding CO_2_, protons, chloride ions and organo-phosphates. The allosteric interaction between O_2_ and H^+^ binding sites is further modulated by organic phosphates that bind preferentially to the T structure and decreases O_2_ affinity.

Since Hb is located inside the RBC, the erythrocyte pH is the true influential factor. In the steady state, both NHE and K^+^-Cl^−^ cotransport are silent and the RBC resembles a Donnan system. When tissue O_2_ consumption increases, capillary P_O2_ decreases and more O_2_ is extracted from the blood. The presence of CAII secures rapid CO_2_ hydration and RBC acidification in tissue capillaries (Bohr shift). H^+^ is bound to Hb, whereas HCO_3_^−^ is transported to plasma in exchange for Cl^−^.

The role of the Hb oxy-deoxy conformational change in securing adequate O_2_ delivery may extend beyond the classical Bohr effect. In recent research, it has been suggested that RBCs play a role in local blood flow regulation by sensing O_2_ demand and matching the release of vasodilatory compounds (Barvitenko et al. 2004; Robinson and Lancaster 2005; Han and Liao 2005). The nature of potential O_2_ sensors (Hb, heme-containing proteins, reactive O_2_ species, and changes in redox status) in erythrocytes remains unclear (Gibson et al. 2000).

## Erythrocyte NHE

NHE is an oxygen-sensitive, electroneutral, secondary active transport, that catalyses amiloride sensitive exchange of one Na^+^ for one H^+^ under the influence of hormones and growth factors (Grinstein and Rothstein, 1986; Bourikas et al. 2003). Intracellular pH is an important modulator of cell function since many enzymes exhibit pH dependence in the physiological range. Until now, pHi was assumed to be spatially uniform. However, recent studies suggest the possibility of spatial nonuniformity of pHi. There could be pHi microdomains, just beneath the plasma membrane, where pHi is tightly regulated and modest fluctuations in pHi arising during physiological cellular activity may influence the function of adjacent proteins (Willoughby et al. 2005; Ro and Carson, 2004). Nonuniform membrane distribution of NHE has been observed: it has been localized to lipid rafts or caveolae along with Ca^++^-sensitive adenylyl cyclases. According to the model of “transport metabolons” (such as co-localised CAII-NHE and CAII-Na^+^-HCO_3_ cotransporter), H^+^ ions generated by CAII activity may be *channelled* directly to the NHE for export, thus creating an alkaline pH microdomain. The dimensions and pH differentials of alkaline and acidic microdomains will depend on the relative production rates of HCO_3_^−^ and H^+^ ions by CAII, the export rates of H^+^, and HCO_3_^−^ by NHE and anion exchanger, respectively, and the diffusion rates of HCO_3_^−^ and H^+^ ions in the cytoplasm. An acidic cluster of amino acid in the cytoplasmic C-terminal region of NHE mediates interaction with the basic N-terminal region of CAII, creating a transport complex or metabolon to facilitate H^+^ export (Li et al. 2006).

NHE1 is a redox-regulated gene (Akram et al. 2006). Reactive oxygen species regulate NHE1 gene expression: O_2_^−^ anion induces NHE1 gene promoter activity resulting in increased NHE-1 protein expression, which correlates with the resistance of cells to death stimuli. In contrast, hydrogen peroxide suppresses NHE1 promoter activity and increases cell sensitivity to death triggers. These findings have important clinical implications on account of the observed erythrocyte NHE hyperactivity in relation to chronic states of oxidative stress in humans (Matteucci and Giampietro, 2000a; Matteucci and Giampietro, 2001; Matteucci and Giampietro, 2006; Matteucci and Giampietro, 2007a and b). Indeed, RBC NHE activity has been noted as being significantly higher in type 1 diabetes (T1D) family members, independently of the presence of renal disease. Moreover, we found evidence of increased oxidative stress in the same nondiabetic normotensive relatives of T1D patients, apart from soluble markers of autoimmunity and despite seemingly intact antioxidant defences. Markers of oxidation were associated with markers of inflammation and we concluded that the familial increase in NHE activity could be ascribed to the direct stimulatory effect of oxidative stress.

## Voltage-Dependent Anion-Selective Channel 1 (Vdac1) between Mitochondria and Cytosol

VDAC1 (or porin) is a 30–35 kDa protein originally discovered in the outer membrane of mitochondria where it is the major pathway for metabolite (ADP/ATP, succinate, citrate) exchange between the cytosol and the mitochondria (Komarov et al. 2005). VDAC anchors both pro- and antiapoptotic proteins and could be part of the cytochrome c release channel in the outer membrane (intrinsic pathway of apoptosis). Predicted structural studies of human VDAC suggest the pore formed from a single layer of protein consisting of one α-helix and either 13 or 16 membrane-spanning strands, curved into a cylinder (Baker et al. 2004). In the closed state, VDAC is slightly cation-selective; in the high conductance configuration (open state) it shows preference for anions (Rostovtseva et al. 2005). Transition between the open and closed states is voltage-dependent. At low voltages (<30 mV) the positively charged voltage sensor forms part of the channel wall, at high voltages it moves out of the lumen causing channel closure (Lemasters and Holmuhamedov, 2006).

Although considered localized exclusively in the outer membrane and at contact sites of mitochondria, VDAC has also been found in the plasma membrane of most tissues, where it is concentrated in caveolae and caveolae-related domains (Bàthori et al. 1999). The presence of VDAC on erythrocyte plasma membrane has been confirmed by using anti-VDAC antibodies (Shimizu et al. 2001). The function of porin in the plasma membrane remains unknown. Beyond known interactions with enzymes and apoptotic modulators, VDAC acts as an anchoring protein and may interact with several cytoskeletal elements (Roman et al. 2006).

## Erythrocyte Plasma Membrane Oxidoreductases

Plasma membrane oxido-reductases (PMORs) are transmembrane electron transport systems that have been found in the membranes of all cells and have been extensively characterized in the human erythrocyte (Kennett and Kuchel, 2006). Erythrocyte PMORs transfer reducing equivalents from intracellular reductants to extracellular oxidants, thus their most important role seems to be to enable cell response to changes in intra- and extracellular redox environments. Putative intracellular sources of reducing equivalents are NADH, NADPH, ascorbate, GSH, flavonoids. Since traditionally PMOR activities were named after the oxidant used to identify them and the supposed intracellular electron donor, there is some confusion on how many PMORs exist (Lawen et al. 2005). The electron acceptor predominantly used to study PMORs is the membrane-impermeable ferricyanide that is reduced to ferrocyanide. The activity of these systems is strictly dependent on cell metabolic status and CO_2_ production through the oxidative pentose phosphate pathway. This activity was increased four-fold above controls in the presence of ferricyanide (May et al. 1996). The association of GE into multimeric complexes near cdb3 suggests a link between PMOR activity and metabolic control. As glycolytic ATP can be channelled to membrane ion pumps, reducing equivalents could be diverted from the glycolytic pathway across the membrane. Renegade electrons could be transported as NADH to the plasma membrane and extruded by the PMOR activities. Whenever PMOR function is perturbed, the production of reactive species may increase. A 35 kDa NADH:ferricyanide reductase has been purified from human plasma membrane preparations and identified as VDAC1 (Baker et al. 2004). A dual role for the plasma membrane VDAC1 has been proposed, i.e. maintenance of cellular redox homeostasis in normal cells and cell volume regulation in apoptotic cells. The expression of functional VDAC activity has been induced by apoptotic stimuli in the plasma membrane of neuronal cell lines, and blocking this activity prevented apoptosis (Elinder et al. 2005).

## Potential Role of Erythrocyte PMORs as Redox Biomarker in Disease States

Despite increasing evidence in literature that PMORs in the plasma membrane may have several functions, little data exists about the erythrocyte electron transfer systems in humans. Ferricyanide reductase activity in a clinical setting was studied for the first time in 1979 (Orringer and Roer, 1979). *Chronic dialysis* patients had higher pre-dialysis ferricyanide reductase activity than controls. However, the accelerated ferrocyanide generation was attributed to a “plasma factor”, probably identified with ascorbic acid, since patients were given 500 mg of ascorbic acid daily. Recently, the erythrocyte velocity of ferricyanide reduction (RBC vfcy) has been reconsidered in uremic patients (not receiving daily vitamin C supplements) as well as the changes induced by a single hemodialysis session (Matteucci and Giampietro, 2007b). Pre-dialysis RBC vfcy was maintained in the normal range on account of a reduced content of GSH and in spite of low plasma ascorbate. A single hemodialysis treatment reduced biomarkers of protein and lipid oxidation but markedly impaired transmembrane electron transfer, which could be explained by acute depletion of electron donors. Indeed, immediately after the dialysis session RBC vfcy acutely and transiently decreases by about 30%, and it occurs concomitantly with a further marked reduction in plasma vitamin C concentration (by about 64%).

In patients with *type 1 diabetes* (T1D) and *diabetic nephropathy* RBC vfcy was peculiarly elevated (Matteucci and Giampietro, 2000b). The abnormality was not contributed to by familiar or hereditary components (electron flow was normal in their non-diabetic siblings) but was suggestively attributed to chemical changes in the intervening medium. Theoretically, the magnitude of the current (I_i_) across membrane ion channels depends on the density of channels (N), the conductance of the open channel (γ(V)), and its open probability (P_o_(V)) (Bezanilla, 2000). The conductance of one open channel γ(V) is constant unless there are extremely asymmetrical ionic conditions or a voltage-dependent block. Thus, the nonlinear dependence of I_i_ with voltage is the result of the modulation of the open probability P_o_ of the channel by voltage. Functionally, there must be a device that detects the voltage across the membrane (Song et al. 1998). The voltage sensor could be a charged domain that moves along the electric field, or a dipole that reorientate with respect to the electric field. For VDAC’s gating processes, decreasing the positive charge on the molecule, decreases the voltage-dependence and vice versa.

Noted in this respect, is the following:

- massive protein oxidation occurs in vivo, particularly in diabetes mellitus and in nephrotic syndrome (Suzuki et al. 1992; Musante et al. 2006),
- the oxidation of the sulphydryl group of cysteine to cysteic acid makes the surface charge more acidic in the pH range of ionization of this chemical group,
- sulphenic transformation of thiol groups predicts formation of new hydrogen bonds with adjacent aminoacids.

For the above reasons, conformational rearrangements associated with oxidation of plasma membrane in diabetic nephropathy could be associated with deviations from normal channel activity. Additional in vivo factors can greatly influence the voltage-gating of VDAC channels and the resulting open probability will be the weighted sum of their effects (Colombini, 2004).

RBC vfcy has been further characterized in *healthy subjects* and *T1D families* with regard to: (a) the rate of electron transport in relation with body mass and metabolic efficiency, and (b) the modulating effects of diet and lifestyle on erythrocyte electron transfer system (Matteucci and Giampietro, 2006; Matteucci and Giampietro, 2007a). Among healthy controls, individuals with BMI ≤25 kg/m^2^ had lower RBC vfcy in comparison with subjects who were overweight or obese. RBC vfcy correlated positively with two indices of fat body mass (BMI and circulating triglycerides), and negatively with an index of lean body mass (24-hour urinary creatinine excretion). In healthy subjects, RBC vfcy showed a negative association with RBC NHE and plasma malondialdheyde (MDA). To the contrary, among relatives, RBC vfcy did not change with BMI; moreover, it showed a positive association with RBC MDA, negative with RBC GSH. Stepwise multiple regression analyses including lifestyle information found different independent variables to be positively associated with RBC vfcy: daily dietary intake of vitamin C among healthy controls, whereas time spent in regular exercise among relatives. Thus, dietary intake of vitamin C and sporting activities modulated erythrocyte electron transfer efficiency. It is noteworthy that, in the cytosol, ascorbic acid can donate electrons to RBC trans-plasma membrane electron transfer activity. In vitro, ascorbate may become the major electron donor, when cytosol ascorbate concentrations are increased by loading the cells with ascorbate, as well as a selective depletion of erythrocyte ascorbate therefore decreasing the capacity for ferricyanide reduction. Moreover, indirect evidence suggested that regular exercise might improve electron transport efficiency (Matteucci and Giampietro, 2006).

The observation that RBC vfcy correlates negatively with RBC NHE activity deserves comment. The unknown erythrocyte membrane complex involved in the process of electron transfer could be influenced by changes in protonation status of the enzyme. Alternatively, the reduction of ferricyanide could be a non-electrogenic process, unlike the reduction of ascorbate free radical (VanDuijn et al. 2001). In this case, NHE activity may prevent the electron shuttle in the plasma membrane to bind a proton when accepting an electron. A more plausible explanation, however, could be now suggested on the basis of evidence that oxidative stress activates NHE (Svegliati-Baroni et al. 1999; Akram et al. 2006). In the healthy control group, RBC vfcy was negatively associated with at least two markers of oxidative damage, plasma MDA and RBC NHE activity, inasmuch as NHE has been suggested to represent a common mediator of the different effects induced by oxidative stress on cells. Furthermore, positive cooperativity has been found in the pH dependence of ion transport through the monomeric VDAC (Rostovtseva et al. 2000).

**Figure 3:**
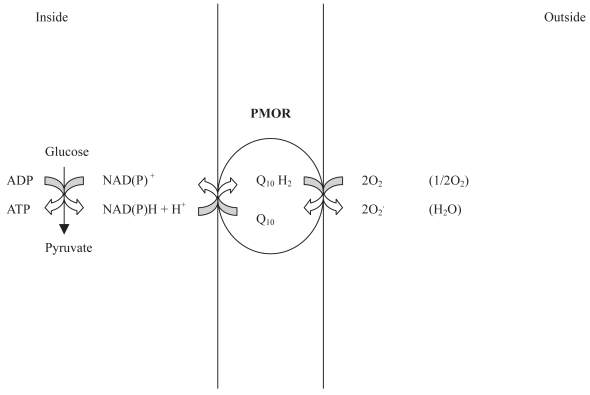
Hypothesis of a complete electron transfer chain in the plasma membrane capable of transferring electrons from NADH to external acceptors via a reduced quinone intermediate. Since oxygen appears to be the natural acceptor, the redox-cycling ubiquinone may transform from a save electron carrier to a superoxide generator if the ubisemiquinone anion becomes protonated.

On the basis of present evidence we propose this scenario:

- In diabetic nephropathy, RBC vfcy is overactive probably due to oxidation-induced changes in protein structure and charge.
- In healthy subjects, erythrocytes supply electrons to reduce extracellular oxidants, thus neutralising their noxious potential. Moreover, according to the model proposed by Morré et al. (2000), the PMOR maintains NAD^+^/NADH homeostasis in mitochondrially deficient cells through the regeneration of oxidised pyridine nucleotide to sustain glycolytic ATP production (Fig. 3).
- In the relatives of T1D patients, vfcy is either positively associated with erythrocyte MDA, i.e. the product of the damaging effect exerted by radicals upon the cell membrane, and negatively associated with the intracellular electron donor GSH. It is plausible that, under conditions of chronically increased radical generation as observed in T1D families, erythrocyte electron transfer may transform from a beneficial electron carrier to a superoxide generator (Fig. 3). As a consequence, the transfer of electrons could result in the generation of ROS at the cell surface (Nohl et al. 1996) capable of propagating the oxidative cascade.

The erythrocyte electron transfer may reflect the functional state of membrane proton pumps that modulate cellular metabolism by regulating the intracellular redox levels. Uncoupling, perhaps as a feed back response to the overproduction of radicals by the electron transport chain, could represent a mechanism for the auto-regulation of radical production, beyond its role in regulating thermogenesis. Recent observation that superoxide increases mitochondrial proton conductance and induces uncoupling (Echtay et al. 2002) supports the hypothesis. The dissipation of the protonmotive force by uncouplers might explain why relatives of type 1 diabetic patients show no relation between vfcy and fat body mass indices.

To conclude; the non uniform distribution of anion exchanger, glycolytic enzymes, NHE, VDAC and CA suggest the existence of wide structural and functional interrelationships among plasma membrane transporters and cytosolic enzymes with unexpected outcomes on cell physiology. The adaptations induced in PMOR function by disorders that affect cell redox state suggest these transmembrane electron transport systems as a potential marker of redox homeostasis. Forthcoming biochemical investigations promise previously unimaginable implications for medical research focused to the comprehension of disease pathophysiology.
